# Facial reactions in response to dynamic emotional stimuli in different modalities in patients suffering from schizophrenia: a behavioral and EMG study

**DOI:** 10.3389/fnhum.2013.00368

**Published:** 2013-07-23

**Authors:** Mariateresa Sestito, Maria Alessandra Umiltà, Giancarlo De Paola, Renata Fortunati, Andrea Raballo, Emanuela Leuci, Simone Maffei, Matteo Tonna, Mario Amore, Carlo Maggini, Vittorio Gallese

**Affiliations:** ^1^Unit of Physiology, Department of Neuroscience, University of ParmaParma, Italy; ^2^Psychiatric Division, Department of Neuroscience, University of ParmaParma, Italy; ^3^Department of Mental Health, AUSL of Reggio EmiliaReggio Emilia, Italy; ^4^Psychiatric Division, Department of Neuroscience, University of GenovaGenova, Italy

**Keywords:** EMG, emotions, empathy, facial mimicry, schizophrenia, simulation

## Abstract

Emotional facial expression is an important low-level mechanism contributing to the experience of empathy, thereby lying at the core of social interaction. Schizophrenia is associated with pervasive social cognitive impairments, including emotional processing of facial expressions. In this study we test a novel paradigm in order to investigate the evaluation of the emotional content of perceived emotions presented through dynamic expressive stimuli, facial mimicry evoked by the same stimuli, and their functional relation. Fifteen healthy controls and 15 patients diagnosed with schizophrenia were presented with stimuli portraying positive (laugh), negative (cry) and neutral (control) emotional stimuli in visual, auditory modalities in isolation, and congruently or incongruently associated. Participants where requested to recognize and quantitatively rate the emotional value of the perceived stimuli, while electromyographic activity of Corrugator and Zygomaticus muscles was recorded. All participants correctly judged the perceived emotional stimuli and prioritized the visual over the auditory modality in identifying the emotion when they were incongruently associated (Audio-Visual Incongruent condition). The neutral emotional stimuli did not evoke any muscle responses and were judged by all participants as emotionally neutral. Control group responded with rapid and congruent mimicry to emotional stimuli, and in Incongruent condition muscle responses were driven by what participants saw rather than by what they heard. Patient group showed a similar pattern only with respect to negative stimuli, whereas showed a lack of or a non-specific Zygomaticus response when positive stimuli were presented. Finally, we found that only patients with reduced facial mimicry (Internalizers) judged both positive and negative emotions as significantly more neutral than controls. The relevance of these findings for studying emotional deficits in schizophrenia is discussed.

## Introduction

Emotional expressions are widely acknowledged as essential in communicating internal feelings and intentions (Ekman and Oster, 1979). The ability to communicate and understand the emotional states of others and their intentions is a fundamental social skill. Indeed, facial expressions are among the most common and significant emotion stimuli.

To this end, it is well-known that humans react to emotional facial expressions with specific, congruent facial muscle mimicry, which can be reliably measured by electromyography (EMG; e.g., Dimberg, 1982, 1988). For example, pictures of sad facial expressions evoke increased muscle *Corrugator Supercilii* activity, while pictures of happy facial expressions increase muscle *Zygomaticus Major* activity and decrease muscle *Corrugator Supercilii* activity (Lundqvist and Dimberg, 1995; Han et al., 2012). These facial muscular reactions appear to be spontaneous and automatic (Dimberg and Thunberg, 1998; Dimberg et al., 2000, 2002; Larsen et al., 2003). Many studies demonstrated that facial mimicry contributes to recognition of specific facial expressions (for a review, see Goldman and Sripada, 2005; Niedenthal et al., 2010). Indeed, blocking facial mimicry impairs recognition of facial expression of emotions (Oberman et al., 2007). Furthermore, it has been proposed that mimicry reflects internal embodied simulation of the perceived facial expression in order to facilitate understanding of its emotional meaning (Gallese, 2003, 2005, 2006; Niedenthal, 2007; Halberstadt et al., 2009; Niedenthal et al., 2009) and promoting empathy by means one's facial feedback system (for a review of the facial feedback hypothesis, see Adelmann and Zajonc, 1989). A recent EMG study (Dimberg et al., 2011) showed that high empathic people, with respect to low empathic group, are particularly sensitive in reacting with facial reactions when they look to emotional facial expressions. Moreover, high empathic people rated perceived facial emotional expressions as more intense with respect to low empathic ones.

Historically, affective features of schizophrenia were considered an integral part of the disorder. Bleuler (1950) considered affective disturbance to be a fundamental symptom of schizophrenia, whereas hallucinations and delusions were regarded as accessory symptoms. Studies on patients' facial mimicry in response to emotional stimuli showed that they activate the same muscle of control subjects, but such activation was found to be weaker in patients than in healthy controls (Earnst et al., 1996; Kring and Earnst, 1999). Another study showed, on the other hand, that in contrast to healthy controls, patients diagnosed with schizophrenia demonstrated atypical facial mimicry, which was not associated with any clinical feature of the disorder. The authors of this study suggested that this evidence might account for a low-level disruption contributing to empathy deficits in schizophrenia (Varcin et al., 2010). Similarly, Wolf et al. (2006) found an undecipherable and bizarre mimic pattern within a sample of patients suffering from schizophrenia, called “*mimic disintegration*” (see Heimann and Spoerri, 1957). Mimic disintegration is defined as the inability to organize specific facial muscle movements as an integrated whole, thus making difficult for observers to decode the emotional state and establish contact or develop a deeper relationship with the patients. Furthermore, many studies investigating everyday life of patients diagnosed with schizophrenia documented an emotional-affective pattern characterized by many negative and few positive experiences, thus making patients' affectivity more negative. Some studies (Mattes et al., 1995; Iwase et al., 1999; Wolf et al., 2004, 2006) found a minor activity of *Zygomaticus* muscle in response to positive stimuli, whereas another study (Sison et al., 1996) found an overall major activation of *Corrugator Supercilii* muscle, interpreted as a sign of the negative attitude showed by patients in everyday life.

Reduced emotional expression (i.e., flat affect) is not only a typical symptom of full-blown schizophrenia (Andreasen, 1984a; Bleuler, 1950). Many findings lend support to the assumption that vulnerability to schizophrenia may be subtly manifested in emotional behavior long before the onset of clinical symptoms. Furthermore, after schizophrenia onset, flat affect increases (Walker et al., 1993). Reduced emotional facial expression could be a disease risk index for high-vulnerability subjects (e.g., Schizotypal Personality patients and first degree relatives) (Phillips and Seidman, 2008). Moreover, previous research on flat affect showed a disjunction between the expression and the experience of emotion in schizophrenia (Bleuler, 1950; Berenbaum and Oltmanns, 1992; Kring et al., 1993; Kring and Neale, 1996; Aghevli et al., 2003; Kring and Earnst, 2003). These studies showed that patients with schizophrenia often reported experiencing strong emotions, but they were significantly less expressive than controls. Thus, observers could note no visible sign of emotion.

The studies using EMG recording to investigate emotional expression in schizophrenia, used different materials and methods. In particular, often non-ecological stimuli, like static images non-facial stimuli, or fiction movies were used. Many studies indeed highlighted, on the other hand, the importance of dynamic stimuli in the evaluation of emotional expression. A recent study on healthy individuals showed that presentation of dynamic facial expressions evokes stronger EMG responses than static ones. Moreover, participants rated dynamic expressions as more intense that static ones (Rymarczyk et al., 2011).

Emotional facial expression communicates feelings, but is also an important low-level mechanism contributing to the experience of empathy, thereby lying at the core of social interaction. Schizophrenia is associated with pervasive social cognitive impairments that include emotional processing of facial expressions. Despite such disorder might play a crucial role in empathizing deficits and consequently impoverished social skills, previous research on facial expression of emotions in schizophrenia has not yielded unequivocal results. In particular, it remains unaddressed the issue of patients' facial expression as a medium of empathic resonance contributing to the recognition and evaluation of the perceived emotion expressed by others.

The aim of this study was to investigate whether subjective facial mimicry affects the quantitative evaluation of the emotional content of perceived emotions presented through dynamic expressive stimuli, in healthy participants and in patients diagnosed with schizophrenia. To this purpose we employed a novel paradigm by means of which emotional dynamic ecological stimuli were presented in the visual and auditory modalities in isolation and congruently or incongruently associated. This approach enabled us to study the dimensional quality and possible alteration of the emotional responses in these two experimental groups.

## Materials and methods

### Participants

Thirty participants took part to the experiment. Control participants (CNT; ten males, five females, mean age 35.8 years *SE* ± 2.3) were recruited by public announcement and were blind to the experimental goals. None of them reported the presence of any neurological or psychiatric disorder. Patient group (SZP; ten males, five females, mean age 32.8 years *SE* ± 1.7) were recruited from the Clinical Psychiatry Institute of the University of Parma. All of them were chronic clinically stable outpatients, mainly diagnosed with schizophrenia, paranoid subtype. Only one patient was diagnosed with a disorganized subtype, one with an undifferentiated subtype and two patients with a residual subtype. Psychiatric diagnosis was established via a structured interview (Structured Clinical Interview for DSM–IV, SCID). Exclusion criteria were the presence of neurological and vascular disorders, dysmetabolic syndrome, alcohol or drugs abuse and mental retardation (Intelligence Quotient score <70). All participants had normal or corrected to normal vision. In addition to being closely matched for gender, the two groups did not differ in age [*t*_(30)_ = −1.06, *p* > 0.05]. All clinical participants (SZP) were receiving antipsychotic medication (most of them were administered new generation atypical antipsychotics). Since the age of onset and the illness duration indicated that the clinical sample was heterogeneous, for comparing dosages of different drugs we converted doses of medication to chlorpromazine equivalents. Then we multiplied these equivalents by the time an individual had been on a given dose to obtain cumulative value measured in dose-years. After each dose had been converted to dose-years, the results could be summed to provide a cumulative measure of lifetime exposure (Andreasen et al., 2010).

In order to describe psychopathological features related with schizophrenia, patients were administered a variety of tests: scale for the Assessment of Negative Symptoms (SANS; Andreasen, 1984a), Scale for the Assessment of Positive Symptoms (SAPS; Andreasen, 1984b), Social Anhedonia Scale (SAS; Chapman et al., 1976), Physical Anhedonia Scale (PAS; Chapman et al., 1976). Given that all patients were under medication, we also administered them the Simpson-Angus Extrapyramidal side-effects Scale (Simpson and Angus, 1970), an established, valid and reliable instrument for assessing neuroleptic-induced parkinsonism (Janno et al., 2005). None of them were beyond cut-off value, indicating that SZP participants did not show any significant extrapyramidal side-effect related with drugs assumption. Details about CNT and SZP samples are provided in Table 1. Written informed consent was obtained from all participants before entering the study. The local Ethical Committee approved the study.

**Table 1:** **Demographic variables and characteristics of Schizophrenia (SZP) and Control (CNT) participants**.

**Characteristic**	**Patients**				**Controls**		
	**Mean**	***SE***	**Range**	**Cut-off**	**Mean**	***SE***	**Range**
Age (years)	32.80	1.69	25–49		35.80	2.28	25–53
SAPS	26.67	4.17	0–170				
SANS	48.09	4.56	0–125				
PAS	26.36	2.26	0–61	>18			
SAS	19.00	1.74	0–40	>12			
Simpson-Angus Scale	0.36	0.04	0–4	>0.65			
Duration of illness (years)	11.23	1.30	2–24				
Age at first psychosis	22.69	0.66	19–28				
Number of hospitalizations	3.83	0.38	2–7				
Dose of typical and atypical antipsychotics	32.85	4.93					
Dose of atypical antipsychotics	24.84	4.00					
Dose of typical antipsychotics	8.01	1.48					

Drugs are expressed as the cumulative value measured in dose-years in the form of (chlorpromazine equivalent in mg) × (time on dose measured in years) (Andreasen et al., 2010).

### Stimuli

Two professional actors (one male and one female) were used for stimuli preparation. Stimuli consisted of 2-s colored video clips showing positive (laugh), negative (cry) and neutral (control) emotions. The neutral video clips showed actors making various faces (i.e., “making a face”) that did not imply any particular emotional content, just that the actors were adopting some specific facial expressions. Actors when performed neutral stimuli always associated the making a face with specific vocalizations. The sound of the neutral stimuli was a vocalization similar to “ahh,” “ohh,” or “eemmh.” Actors' full face was presented against a gray background. Stimuli consisted of actors' Laugh (Positive), Cry (Negative) and Control (Neutral) accompanied by the simultaneously produced sound of laughter, crying and a non-emotional sound, respectively. Half of the stimuli was performed by the male actor, whereas the other half was performed by the female actress. Stimuli were recorded using a digital camera (25 frames/s, 720×576 pixels), with audio digitally recorded at 44.1 kHz. Stimuli were divided into four presentation modalities: Visual only, Audio only, Audio-Visual congruent and Audio-Visual incongruent. Every presentation modality was made of 60 stimuli [24 Laugh (Positive) stimuli, 24 Cry (Negative) stimuli and 12 Control (Neutral) stimuli]. In the Audio modality (A), the sound of the video clips of laugh, cry and control stimuli was extracted from the original video clips and presented alone. In the Video modality (V), only the visual component of video-clips was presented, devoid of any sound. In the Audio-Visual Congruent modality (AVC), the original video clips were presented with both modalities. In the Incongruent Audio-Visual modality (AVI), the video of a given expression was coupled and presented with the audio pertaining to a different video clip performed by the same actor (e.g., audio of laugh with the video of cry, audio of cry with the video of laughs and audio of a given neutral sound with the video of another neutral stimulus). Consequently, in AVI Laugh participants saw an actor crying but heard laughing, in AVI Cry participants saw an actor laughing but heard crying, and in Control condition they saw an actor making an unemotional face while hearing the sound of a different neutral stimulus.

### Experimental procedure

Participants were individually tested in a sound attenuated laboratory room. They were invited to sit on a comfortable chair in front of a 19-inch computer monitor used for stimuli presentation, located at a distance of 70 cm. Audio tracks were presented at a comfortable sound level (<70 dB) through loudspeakers integrated in the computer monitor. Before starting, participants were invited to relax and refrain from moving during the experiment. Participants were instructed to carefully listen to and/or watch audiovisual stimuli. After exposure to each stimulus, participants were required to verbally rate how much positive or negative the stimulus was perceived on a Likert scale ranging from −3 (very negative) to +3 (very positive), where 0 indicated lack of perceived emotional content.

**Figure 1:**
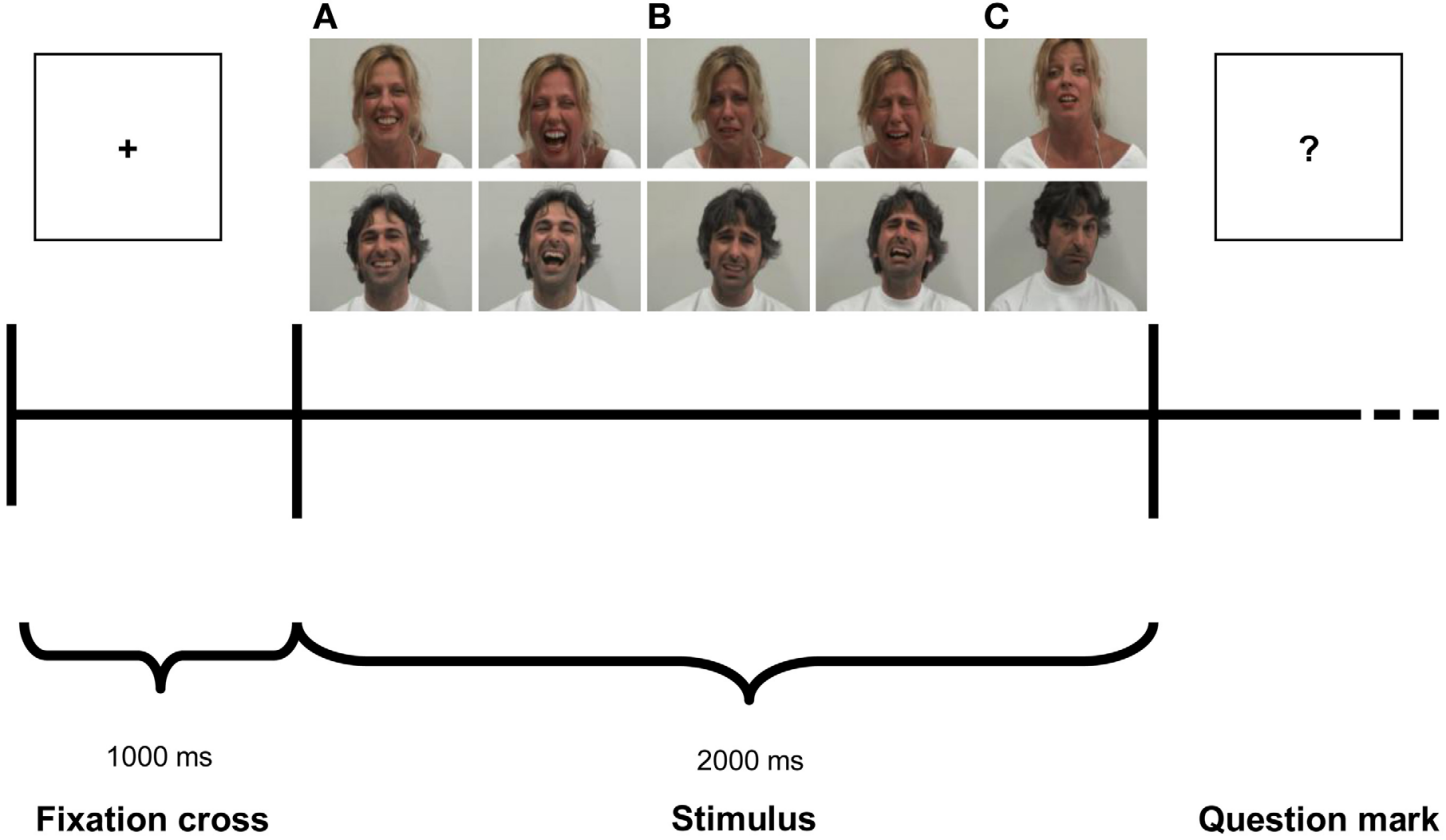
**Experimental paradigm**. Photographs illustrate examples of stimuli depicting Laugh **(A)**, Cry **(B)** and Control **(C)**.

The experiment consisted of four experimental blocks of 60 stimuli each presented in randomized order. Each block consisted of one of the four modalities: Audio-Visual Congruent (AVC), Audio-Visual Incongruent (AVI), Audio (A) and Video (V). In every modality three emotional stimuli were presented in randomized sequence: Laugh (Positive), Cry (Negative) and Control (Neutral). A pause was provided at the end of each condition. The order of blocks was counterbalanced among participants. Each trial (Figure 1) started with a fixation cross (the “+” symbol) presented for 1000 ms (baseline), immediately followed by the stimulus, which lasted 2000 ms, then followed by a question mark (the “?” symbol). After question mark presentation, participants verbally scored the emotional valence of each stimuli. The experimenter took note of participants' response in a record sheet and then started manually the next trial. The total duration of the experiment was about 40 min.

### EMG recording

To measure facial muscle activity, Ag/AgCl surface electrodes (diameter 3 mm) were attached bipolarly over the left (Dimberg and Petterson, 2000) *Zygomaticus major* and the *Corrugator Supercilii* muscle regions (Fridlund and Cacioppo, 1986). In order to reduce the inter-electrode impedance, the participants' skin was cleaned with alcohol and rubbed with the electrode paste. Continuous electromyography (EMG) recordings from both muscles were simultaneously acquired with a CED Micro 1401 analog-to-digital converting unit (Cambridge Electronic Design, Cambridge, UK). The EMG signal was amplified (3000×), digitized (sampling rate: 2.5 kHz) and stored on a computer for offline analysis.

### Data and statistical analysis

#### Behavioral rating

The rating score of each participant was averaged on the basis of modality and emotion. The corresponding averaged rating scores were entered into a 4 (Modality: AVC, AVI, A, V) × 3 (Emotion: Laugh, Cry, Control) × 2 (Group: SZP and CNT) repeated measures ANOVA, with Modality and Emotion as within-participants factors and Group as between-participants factor.

#### EMG data analysis

Offline, data were submitted to a 50–500 Hz band-pass filter to reduce movement related artifacts and environmental noise, and full-wave rectified. Data were then visually inspected, and data with remaining artifacts were excluded from subsequent analysis [mean percentage of discarded trails: 14.1% for CNT, 12.6% for SZP; *T*-test performed did not show significant differences between groups *t*_(30)_ = 0.5, *p* > 0.6]. In accordance with earlier experiments (e.g., Dimberg et al., 2000), any distinct muscle response to the stimuli was expected to be detectable after 500 ms of exposure. Thus, for each participant and trial, the averaged EMG responses of the two muscles were subdivided in 4 time periods (T1–T4) of 500 ms each. Each time-bin was then normalized with respect to the baseline (i.e., averaged pre-stimulus signal activity lasting 500 ms: from 250 to 750 ms of the 1000 ms total duration of the baseline). Thus, an EMG normalized value above the 100% means an activation of a given muscle with respect to the baseline, whereas an EMG normalized value below the 100% indicate a relaxation of that muscle with respect to the baseline. In order to compare baselines, we performed two ANOVAs, one for each muscle, in which baselines raw data were compared, with Modality (AVC, AVI, A, V) as within-participants factor and Group (SZP, CNT) as between-participants factor. Mean EMG responses were then calculated for each Modality (AVC, AVI, A, V), Emotion (Laugh, Cry, Control) and Period (T1, T2, T3, T4). EMG data were entered into a 4 (Modality: AVC, AVI, A, V) × 3 (Emotion: Laugh, Cry, Control) × 4 (Period: T1: 0–500 ms, T2: 500–1000 ms, T3: 1000–1500 ms, T4: 1500–2000 ms) repeated measures ANOVA, with Modality, Emotion and Period as the within-participants factors and Group (SZP and CNT) as between-participants factor. One separated ANOVA was conducted for each muscle (Corrugator and Zygomaticus).

#### Functional relation between EMG and behavioral rating

In order to investigate functional relations between the recorded EMG responses and behavioral rating, we calculated median EMG responses, separately for each group and for each emotion (positive, negative), irrespective of modalities and periods. We excluded from this analysis Control stimuli because they did not evoke any significant EMG response in both muscles (see Results). Regarding positive emotions, we considered for this analysis the following modalities in which we measured (see Results) Zygomaticus muscle activation: AVI Cry, AVC Laugh, A Laugh and V Laugh. Regarding negative emotions, we considered the following modalities in which we measured (see Results) Corrugator muscle activation: AVI Laugh, AVC Cry, A Cry and V Cry.

For each participant, we calculated the median EMG response for each emotion (positive, negative). If this value was equal or greater than the median value calculated separately for positive and negative emotions for the group the participant belonged to, we classified this participant as Externalizer. If, instead, this value was smaller than the median value calculated separately for positive and negative emotions for the group the participant belonged to, we classified this participant as Internalizer (see Kring and Gordon, 1998). Following this procedure, in the CNT group we obtained the median value of 95.14% (8 Externalizers and 7 Internalizers) for positive emotions and the median value of 99.24% (8 Externalizers and 7 Internalizers) for negative emotions. In the SZP group we obtained the median value of 95.15% (6 Externalizers and 9 Internalizers) for positive emotions and the median value of 100% (6 Externalizers and 9 Internalizers) for negative emotions. The corresponding averaged rating scores were entered into a 4 (Modality: AVC, AVI, A, V) × 2 (Group: SZP and CNT) repeated-measures ANOVAs, with Modality as within-participants factor and Group as between-participants factor. Overall, we ran totally 4 ANOVAs, two in order to analyze behavioral data of the Externalizer cohort (one for each emotion valence: positive, negative) and two in order to analyze behavioral data of the Internalizer cohort (one for each emotion valence: positive, negative).

For all performed analyses, the significance level was set at *p* < 0.05. *Post-hoc* comparisons (LSD Fisher test) were applied on all significant main factors and interactions.

## Results

### Behavioral results

**Figure 2:**
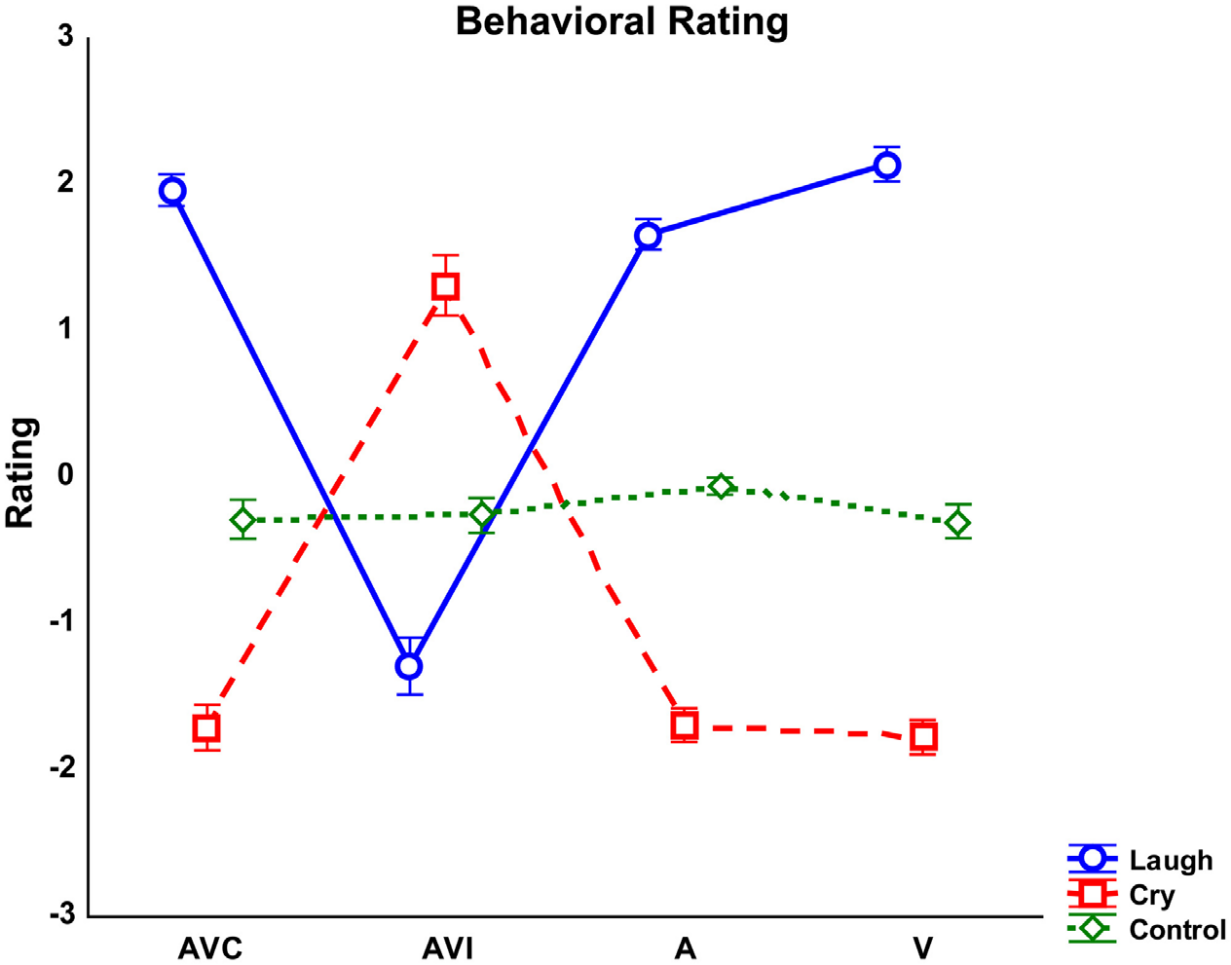
**Averaged rating scores detected for each modality (AVC, Audio-Visual Congruent; AVI, Audio-Visual Incongruent; A, Audio; V, Video) and emotion (Laugh, Cry, Control)**. Error bars represent standard errors of mean (*SE*).

Results of the repeated-measures ANOVA performed on behavioral rating scores showed that the factor Emotion was significant [*F*_(2, 56)_ = 222.52 *p* < 0.000]. *Post-hoc* comparisons showed that Cry was rated by both groups more negative than Laugh and Control stimuli were considered without any emotional content (all *p*_s_ < 0.000). Complementing this finding, the interaction between Emotion and Modality [*F*_(6, 168)_ = 156.7 *p* < 0.000] was also significant (Figure 2). This interaction was due to the fact that the negative rating scores reported for Laugh stimuli during the AVI modality (in which participants saw cry and heard laugh) differed from the positive rating scores reported for Laugh stimuli in all other modalities (all *p*_s_ < 0.000). Similarly, the positive rating scores reported for Cry stimuli during the AVI modality (in which participants saw laugh and heard cry) significantly differed from the negative rating scores reported for Cry stimuli in all other modalities (all *p*_s_ < 0.000). These differences in rating of AVI modality were due to the fact that both groups based their ratings on the emotion they saw (Laugh in AVI Cry and Cry in AVI Laugh), instead of the emotion they heard. *Post-hoc* analysis also showed that Control stimuli were rated as devoid of emotional content in all modalities by both groups (all *p*_s_ < 0.000). Furthermore, with Laugh stimuli, V modality was rated more positive than A modality (*p* < 0.05).

Results also showed a significant interaction Emotion by Group [*F*_(2, 56)_ = 3.43 *p* < 0.05]. However, *post-hoc* analyses revealed no significant differences between groups (all *p*_s_ > 0.3).

### EMG results

Two repeated measure ANOVAs, one for each muscle (Corrugator, Zygomaticus), were performed in order to compare baselines between the two groups, with Modality (AVC, AVI, A, V) as within-participants factor and Group (SZP, CNT) as between-participants factor. We found no significant main effect and interactions (all *p*_s_ > 0.05). These results show that the baselines were not significantly different between the two groups.

**Figure 3:**
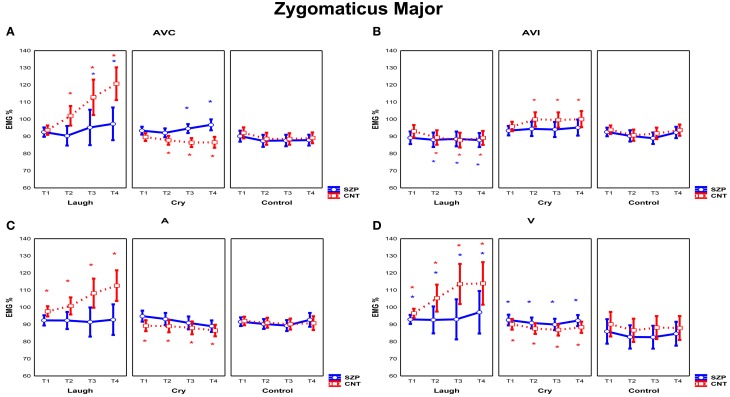
**Mean EMG responses recorded for *Zygomaticus Major* muscle for each modality [AVC (Audio-Visual Congruent) (A), AVI (Audio-Visual Incongruent) (B), A (Audio) (C), and V (Video) (D)]**. The significant differences are indicated by colored asterisks (red for CNT, blue for SZP). Asterisks located in the upper part of the panels indicated a significant activations with respect to Control stimuli; asterisks located in the lower part of the panels indicated significant differences between emotions. Y-axis: 100% represents the mean EMG response of the baseline. X-axis: Time Periods (T1: 0–500 ms, T2: 500–1000 ms, T3: 1000–1500 ms, T4: 1500–2000 ms). Error bars represent standard errors of mean (*SE*).

**Figure 4:**
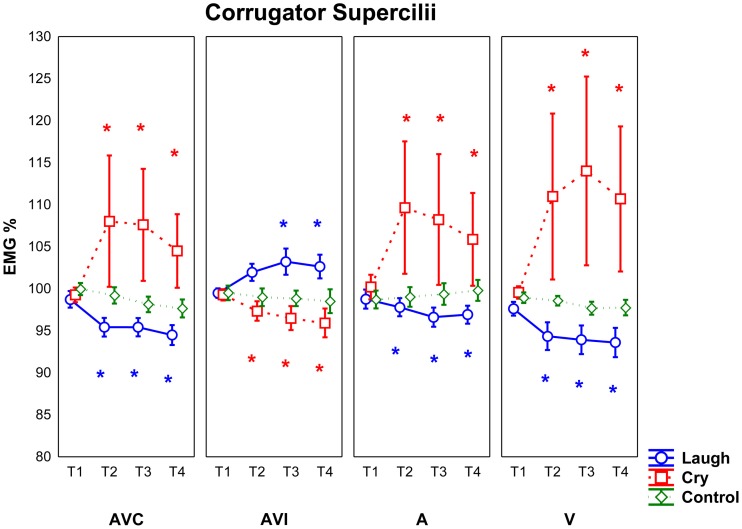
**Mean EMG responses recorded for *Corrugator Supercilii* muscle for all participants (SZP, CNT)**. All other conventions as in Figure 3.

Two repeated measures ANOVAs were performed in order to assess *Zygomatic Major* and *Corrugator Supercilii* EMG responses during the presentation of the stimuli of positive, negative and neutral facial expressions and/or related sounds in four different modalities (AVC, AVI, A, V) (See Figures 3, 4).

#### Zygomaticus major muscle

The analysis of *Zygomaticus Major* muscle EMG responses revealed a significant main effect of Emotion [*F*_(2, 56)_ = 4.20 *p* < 0.05]. *Post-hoc* showed that during the presentation of Laugh stimuli EMG responses were stronger than during the presentation of Control stimuli (*p* < 0.01).

Furthermore, the interaction Modality by Emotion was also significant [*F*_(6, 168)_ = 4.55 *p* < 0.001]. *Post-hoc* showed that in AVI Cry condition (in which participants saw an actor laughing but heard crying) EMG Zygomaticus responses were stronger with respect to A cry and V cry (*p* < 0.05). In AVI Laugh condition (in which participants saw instead an actor crying but heard laughing), EMG Zygomaticus responses were weaker than to all other conditions (i.e., AVC laugh, A laugh and V laugh) (*p* < 0.01). In sum, results showed that EMG Zygomaticus responses in AVI modality were driven by what participants saw and not by what they heard. Furthermore, *post-hoc* analysis revealed that EMG responses were not modulated in all different modalities by Control stimuli presentation (all *p*_s_ > 0.05). The interaction Emotion by Period was also significant [*F*_(6, 168)_ = 2.82 *p* < 0.05]. *Post-hoc* comparisons showed that during Laugh stimuli presentation Zygomatic EMG responses increased with time (T1 vs. T3 *p* < 0.01, T1 vs. T4 *p* < 0.0001, T2 vs. T4 *p* < 0.01), whereas no modulation through time periods was found during Cry and Control stimuli presentation (all *p*_s_ > 0.05).

The interaction Modality by Emotion by Period was also significant [*F*_(18, 504)_ = 3.19 *p* < 0.0001]. Of most interest, a significant interaction of all factors was observed among Modality, Emotion, Period and Group [*F*_(18, 504)_ = 1.68 *p* < 0.05] (Figure 3). Since Control stimuli were rated by both groups (SZP, CNT) as neutral and EMG activity was not modulated during perception of these stimuli (all *p*_s_ > 0.1), we can considered them as effective neutral stimuli for emotion perception. Thus, we compared EMG Zygomaticus activity during the presentation of positive (Laugh) and negative (Cry) emotion-related stimuli with neutral (Control) stimuli.

In line with previous literature (Dimberg and Thunberg, 1998), *post-hoc* comparisons revealed that CNT group showed Zygomaticus EMG responses when they saw and heard actors laughing in a congruent way (i.e., AVC modality) 500 ms after stimulus onset (T2, T3, T4; all *p*_s_ < 0.000). By comparing EMG responses to positive and negative stimuli (Figure 3A), we found an inhibition in the same temporal periods for the latter ones (T2, T3, T4; all *p*_s_ < 0.000). During perception of positive stimuli SZP group showed significant EMG activation, occurring later, 1000 ms after stimulus onset (T3, T4; all *p*_s_ < 0.01). However, the same EMG responses were recorded during both positive and negative stimuli presentations (T3, T4; all *p*_s_ > 0.8). We thus defined this EMG response as “non-specific activation,” because it appeared independently of the perceived emotion (Laugh, Cry).

As shown in Figure 3B, in CNT participants Zygomaticus EMG responses occurred when they saw actors laughing but they heard crying (i.e., AVI Cry condition, in which the visual and auditory components of the stimuli are combined in an incongruent way) 500 ms after stimulus onset (T2, T3, T4; all *p*_s_ < 0.05). By comparing EMG activity recorded during AVI Laugh condition with that recorded during AVI Cry condition, we found inhibition in the same time periods (T2, T3, T4 all *p*_s_ < 0.001). In AVI condition, we thus observed that Zygomatic muscle activation was driven by what CNT saw and not by what they heard.

SZP participants did not activate Zygomatic muscle in this condition (all *p*_s_ > 0.05). By contrasting EMG activity during AVI Laugh condition with that recorded during AVI Cry condition, inhibition was found (T2, T3, T4; all *p*_s_ < 0.05). When emotional visual and auditory information contrasted, patients did not activate EMG Zygomaticus muscle.

As shown in Figure 3C, in CNT group Zygomaticus EMG responses occurred when they only heard laughing (i.e., A modality) already at T1 that is, before 500 ms from stimulus onset (T1, T2, T3, T4; all *p*_s_ < 0.05). By comparing EMG activity recorded during Cry stimuli with that recorded during Laugh stimuli, inhibition during the same time periods was found (T1, T2, T3, T4; all *p*_s_ < 0.01).

SZP participants did not activate Zygomatic muscle in this condition (all *p*_s_ > 0.4). When positive emotional auditory information was presented alone, patients did not react with any Zygomatic EMG responses.

As shown in Figure 3D, in CNT group Zygomaticus EMG responses occurred when they only saw laugh (i.e., V modality) already at T1 that is, before 500 ms from stimulus onset (T1, T2, T3, T4; all *p*_s_ < 0.05). Still in V modality, by comparing EMG activity recorded during Cry stimuli with that recorded during Laugh stimuli, inhibition during the same time periods was found (T1, T2, T3, T4; all *p*_s_ < 0.05).

In SZP group EMG activation occurred already at T1 that is, before 500 ms from stimulus onset (T1, T2, T3, T4; all *p*_s_ < 0.05) during positive stimuli. However, no difference was found between EMG responses recorded during negative and positive stimuli, because, similarly to AVC modality, Zygomaticus responded also during negative stimuli presentation (T1, T2, T3, T4; all *p*_s_ > 0.05). We define this EMG response as “non-specific activation,” because it appeared independently of the perceived emotion (Laugh, Cry).

#### Corrugator supercilii muscle

The analysis of *Corrugator Supercilii* muscle EMG responses revealed a significant interaction Modality by Emotion [*F*_(6, 168)_ = 3.11 *p* < 0.01]. *Post-hoc* showed that in AVI Cry condition (in which participants saw an actor laughing but heard crying) EMG Corrugator responses were weaker than in all other conditions (i.e., AVC cry, A cry and V cry) (*p* < 0.05). In AVI Laugh condition (in which participants saw an actor crying but heard laughing), EMG Corrugator responses were stronger only with respect to the V laugh condition (*p* < 0.05). Furthermore, *post-hoc* analysis revealed that EMG responses were not modulated by different modalities during Control stimuli presentation (all *p*_s_ > 0.7).

Most interestingly, a significant interaction Modality by Emotion by Period was also observed [*F*_(18, 504)_ = 2.78 *p* < 0.001] (Figure 4). Since Control stimuli had been rated neutral by both groups (SZP, CNT) and EMG activity was never modulated during this condition (all *p*_s_ > 0.2), we considered Control stimuli, also for Corrugator EMG responses, as effective neutral stimuli for emotion perception. We performed the same comparisons already described for Zygomaticus muscle.

*Post-hoc* comparisons revealed that in both groups (SZP, CNT) Corrugator EMG responses occurred when they saw and heard cry (i.e., AVC modality) 500 ms after stimulus onset (T2, T3, T4; all *p*_s_ < 0.01). Inhibition occurred during positive stimuli presentation in the same time periods (T2, T3, T4; all *p*_s_ < 0.000).

Both groups activated Corrugator muscle when they saw cry but they heard laugh (i.e., AVI Laugh modality) 1000 ms after stimulus onset (T3 *p* < 0.05; T4 *p* = 0.05). By comparing EMG activity recorded during AVI Cry with that recorded during AVI Laugh, inhibition 500 ms after stimulus onset was found (T2, T3, T4 all *p*_s_ < 0.05). In AVI modality, Corrugator muscle activation was driven by what both groups of participants saw and not by what they heard.

Corrugator EMG responses occurred when both groups heard cry (i.e., A modality) 500 ms after stimulus onset (T2, T3, T4; all *p*_s_ < 0.01), as it happened in AVC modality. EMG activity was inhibited during presentation of Laugh stimuli in the same time periods (T2, T3, T4; all *p*_s_ < 0.0001).

Corrugator EMG responses occurred when both groups saw cry (i.e., V modality) 500 ms after stimulus onset (T2, T3, T4; all *p*_s_ = 0.0000), as in AVC and A modalities. EMG activity was inhibited during presentation of Laugh stimuli in the same time periods (T2, T3, T4; all *p*_s_ < 0.000).

For Corrugator muscle, we found no significant main effects and interactions of Group factor (all *p*_s_ > 0.05).

### Functional relations between EMG and behavioral rating

In order to analyze behavioral data of the Externalizer and Internalizer cohorts, each of which comprised patients and control participants, two ANOVAs for each cohort (one for each emotion: positive, negative) were run.

#### Externalizer cohorts

For both the Externalizer cohorts, no significant Group main effects and interactions were found for positive emotions (all *p*_s_ > 0.8) as well as for negative emotions (all *p*_s_ > 0.8). For positive emotions, only a significant main effect of Modality [*F*_(3, 42)_ = 5.13 *p* < 0.01] was found. Since no other significant main effects and interactions were detected, this means that participants belonging to the Externalizer cohorts, more responding with congruent facial mimicry to positive and negative emotions, also gave correct behavioral ratings, with no differences between groups.

#### Internalizer cohorts

Regarding the results of the ANOVA performed on the Internalizer cohort for positive emotions, we found a significant main effect of Group [*F*_(1, 12)_ = 7.40 *p* < 0.05]. *Post-hoc* comparisons revealed that CNT group gave more positive ratings than SZP group (*p* < 0.05). The factor Modality was also significant [*F*_(3, 36)_ = 4.77 *p* < 0.01] and AVI modality received lower ratings with respect to AVC and V modalities (*p*_s_ < 0.01).

Regarding the results of the ANOVA performed on the Internalizer cohort for negative emotions we found, again, a significant main effect of Group [*F*_(1, 12)_ = 5.63 *p* < 0.05]. *Post-hoc* comparisons revealed that CNT group gave more negative ratings than SZP group (*p* < 0.05). The factor Modality was also significant [*F*_(3, 36)_ = 4.44 *p* < 0.01], and AVI modality received the most positive ratings with respect to all other modalities (*p*_s_ < 0.05).

**Figure 5:**
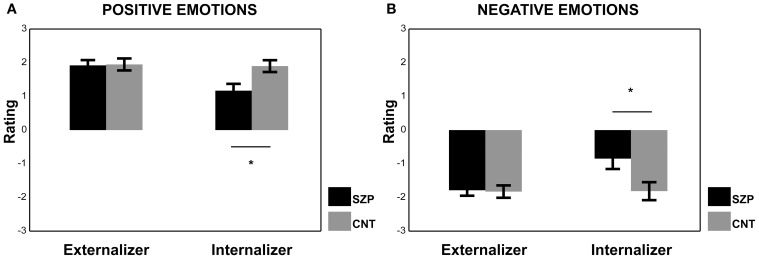
**Mean rating scores of Externalizer and Internalizer cohorts during the presentation of Positive (A) and Negative (B) emotions**. Asterisks represent significant differences between SZP and CNT. All other conventions as in Figure 3.

The Internalizer cohort had EMG below the 100% (i.e., below the baseline value), therefore those participants did not activate their muscles in response to emotional stimuli. Within the Internalizer cohort, we found a significant difference between CNT and SZP groups both for positive and negative emotions. Interestingly, SZP group gave more neutral ratings to perceived positive and negative emotions (see Figure 5).

## Discussion

Our study shows that SZP and CNT participants adequately recognized the emotional quality of the stimuli in all modalities: both groups judged Laugh as positive emotion, Cry as negative emotion and Control stimuli as devoid of any emotional content. Similarly, they were able to score dimensionally the perceived emotions (Laugh, Cry, Control) and, in AVI condition, both groups privileged the visual over the auditory modality. Indeed, they judged the emotion they saw rather than the emotion they heard.

With respect to EMG recordings, CNT group results are coherent with previous studies (Dimberg and Thunberg, 1998) that documented the role of Zygomaticus muscle in response to positive emotional stimuli and that of Corrugator Supercilii in response to negative ones. Also the timing of the activation of the muscles was in line with previous findings (500 ms after stimulus onset). However, with “single modalities” (A and V) intense EMG responses of Zygomaticus muscle were evoked by positive emotions even before 500 ms from stimulus onset. Notably, the inclusion of Control stimuli enriched the experimental paradigm with an effective neutral stimulus, which proved to evoke no EMG response and be judged by all participants as emotionally neutral. This shows that facial mimicry does not occur indiscriminately whenever one looks at the moving face of someone else, but requires the observation of emotion-specific pattern of facial movements. The same emotional specificity of EMG activation also occurred in Audio modality where only Laugh and Cry sounds were able to evoke EMG activation of the Zygomaticus and Corrugator muscles, respectively.

A further innovative feature of the adopted paradigm was the Incongruent (AVI) condition. Interestingly enough, we discovered that in AVI condition healthy participants reacted with rapid and automatic mimicry following the visual emotional content of the stimuli, while disregarding the auditory expressed contrasting emotion.

Whereas CNT and SZP groups reacted to negative emotional stimuli in the same way, SZP participants did not respond or showed inadequate EMG reactions (a “non-specific response”) to positive emotions. Indeed, in AVI and Audio modalities no EMG activation was found, whereas in AVC and V modalities a non-specific response appeared independently of the perceived emotion (Laugh, Cry).

Several studies investigated how emotional stimuli conveyed by visual and auditory modalities are integrated in healthy population (for a review, see Klasen et al., 2012) and in patients with schizophrenia (de Gelder et al., 2005; de Jong et al., 2009, 2010; Castagna et al., 2013). Studies conducted with healthy participants demonstrated that congruent audiovisual emotions usually yield the highest recognition rates, followed by visual emotions, with the auditory emotions being most difficult to classify (Pourtois et al., 2005; Kreifelts et al., 2007; Collignon et al., 2008; Klasen et al., 2011). In our experiment, both groups prioritized the visual over the auditory modality in identifying the emotion when they were incongruently associated (i.e., AVI modality). Furthermore, only for positive emotions, Video modality was rated by all participants as more intense with respect to Audio modality, whereas the AVC modality was judged as equally intense with respect to single modalities (i.e., Audio and Video). The lack of significant rate advantage of bimodality may be explained by a ceiling effect of high rates in both unimodal conditions (cf. de Gelder and Vroomen, 2000).

A recent study investigating multisensory integration in schizophrenia (Castagna et al., 2013) found that patients were not impaired in basic non-emotional and emotional prosody tasks, whereas they showed a specific impairment in decoding emotion in a conflicting auditory condition (i.e., when the content of a sentence was not congruent with the emotional tone expressed by the voice) and in a multisensory integration condition (i.e., when complex emotional auditory and visual cues had to be associated). Another study (de Jong et al., 2010) found that in contrast to controls, a stronger impact of facial on vocal emotion perception occurs in patients diagnosed with schizophrenia. Differently from our experiment, all these studies used complex tasks (e.g., cross-modal emotional recognition tasks) that required top-down attentional processes that demanded superior cognitive abilities (i.e., executive functions), which are notoriously impaired in patients suffering from schizophrenia (Castagna et al., 2013). In our experiment, instead, we investigated automatic bottom-up responses to multimodal emotional stimuli presentation.

In our study, we found that CNT and SZP groups similarly reacted with EMG corrugator responses to negative emotional stimuli. Notably, our findings regarding patients' motor resonance in response to negative stimuli cohere with previous qualitative and quantitative studies documenting how everyday life of patients suffering from schizophrenia is marked by selective biases toward negative emotional experiences which amplify stress-vulnerability and are possibly fostered by persecutory feelings, increased impressionability and oversensitivity to perceived threats. This might be interpreted in line with Kapur's concept of *aberrant salience*, which posits that positive symptoms of schizophrenia may arise out of “the aberrant assignment of salience to external objects and internal representations” (Kapur, 2003; Van Os and Kapur, 2009). Hence patients' emotional susceptibility to negative stimuli, resulting in their persistent negative attitude in everyday life might act as a self-perpetuating mechanism of disturbed salience (Mattes et al., 1995; Kring and Earnst, 1999). Thus, positive daily experiences are few and the occasions of showing congruent motor resonance with happy emotions could be consequently uncommon in patients suffering from schizophrenia (Kring and Earnst, 2003; Wolf et al., 2004, 2006; Trèmeau, 2006), hence the lack of specific and congruent responses of Zygomaticus muscle to positive stimuli.

According to previous results (Kring and Neale, 1996; Sison et al., 1996; Aghevli et al., 2003), patients diagnosed with schizophrenia also showed a disjunction between the expression and the behavioral rating of emotions. In the present study we found this dissociation only for positive emotions, where normal emotional rating was not accompanied by congruent EMG responses.

However, by dichotomizing both groups of participants in two cohorts (Externalizers and Internalizers, Kring and Gordon, 1998) according to the intensity of their EMG congruent responses, we found that the patients' cohort of Internalizers gave more neutral ratings with respect to control group. This means that in patients facial mimicry in response to positive and negative emotions is crucial to correctly judge from a dimensional point of view the perceived emotion. These data cohere with previous findings documenting empathic response deficits in Schizophrenia (Derntl et al., 2009; Varcin et al., 2010) that may be related with abnormalities in the mirror neurons mechanisms. According to this model, involuntary facial mimicry constitutes an important low-level mechanism contributing to the experience of empathy (for a review, see Singer and Lamm, 2009), *via* processes of simulation and perception-action coupling subserved by activation of the mirror neurons mechanism. In other words, involuntary facial mimicry reflects an embodied simulation of the perceived emotion, which facilitates its understanding (Gallese, 2003, 2005, 2006; Niedenthal, 2007; Halberstadt et al., 2009; Niedenthal et al., 2009) by promoting primary empathic resonance on a bodily level (Gallese, 2001; Preston and De Waal, 2002; Sonnby-Borgström, 2002; Sonnby-Borgström et al., 2003; Oberman et al., 2007). Hence, the disruption of this low-level mechanism may contribute to the well-known empathy deficits in schizophrenia (Varcin et al., 2010). Along similar lines, Dimberg et al. (2011) demonstrated that the ability to react with facial EMG activations to facial expressions and to rate these stimuli as more intense is particularly evident among people with high emotional empathy. Our findings cohere with those of Dimberg et al. (2011), since Internalizer patients neither react with any EMG response, nor rated positive or negative emotional stimuli as significantly more intense.

It should be added that Internalizer healthy participants could correctly score perceived emotions despite their apparent EMG hyporeactivity. This result shows that multi-modal emotion recognition can occur even without full-blown facial mimicry. This might be due to the recruitment of high-level cognitive mechanisms possibly fostered through coping strategies. Facial mimicry might be a necessary condition *for fine-grained emotional evaluation* only for Internalizer patients, who are impaired in correctly judging the intensity of positive and negative emotions.

The interpretation of the current findings, however, should be tempered by some limitations. First, the relatively modest sample size reduced the statistical power. Hence possible group differences, such as regarding the EMG corrugator response to negative stimuli, might not have been detected. Second, all participants with schizophrenia were under antipsychotic medications, which might act as a confounders in EMG responses. Nonetheless, since the participants' SAS score (a specific psychometric index sensitive to neuroleptic-induced parkinsonism) was below the cut-off, we are inclined to consider minimal such potential confounder.

It might also be worth noting, that group differences in EMG activation could not be attributed to attentional or motivational factors, for three main reasons. First, all patients were clinically stable (i.e., without hallucinations and similarly flamboyant psychopathology) when underwent the current experiment. Second, the ratings showed that both groups correctly scored the different emotions without significant inter-group differences. Third, the lack of responses that characterized patients was emotion specific, was present only in two modalities, and it was not casually distributed among conditions.

In conclusion, this study provides new evidence on the emotion-specificity of facial mimicry. Further, it demonstrates that (1) congruent facial mimicry can be evoked multi-modally and that (2) when Video and Audio modalities are incongruently associated, the Video modality prevails on the Audio as a response-trigger. The paradigm also proved sensitive to detect deficits in rapid facial mimicry for positive emotions in patients diagnosed with schizophrenia. We interpreted such deficits in rapid facial mimicry as indicative of a possible low-level impairment of motor resonance mechanisms, which may explain a portion of the empathizing deficits in schizophrenia. This coheres with our finding that the weaker facial mimicry response shown by patients' Internalizer cohort is related to difficulties in correctly judging the intensity of positive and negative emotions.

In our view, these findings could lead to future studies on the nature of emotional deficits in Schizophrenia, capitalizing on the convergence between neuroscience and psychopathology. Indeed, contemporary psychopathological research emphasizes the relevance of disruption of implicit bodily functioning (of which facial mimicry is a crucial component) for the loss of practical immersion in the intersubjective world that constitutes the hallmark of schizophrenia spectrum vulnerability (see Parnas et al., 2002; Stanghellini, 2004; Fuchs, 2005; Parnas, 2011; see also Ebisch et al., 2012; Ferri et al., 2012; Gallese and Ferri, 2013). Therefore, the disturbance of motor resonance revealed in this study, might be implicated in some of the disorders of intersubjective attunement that phenomenologically-oriented psychopathology indicates as core features of Schizophrenia (Minkowski, 1927; Blankenburg, 1971; Parnas and Bovet, 1991; Parnas et al., 2002).

The interpretation of these results grounded on the hypothesis of an impaired functionality of motor resonance mechanisms in patients diagnosed with schizophrenia, should be limited by the lack in our study of direct measures of the entailed underpinning neural mechanisms. Nevertheless, a previous fMRI study carried out by Carr et al. (2003), demonstrated that in healthy participants observation and imitation of emotions activated a similar neural network of brain areas, in which the insula acted as an interface between the premotor component of the mirror mechanism and the limbic system, thus enabling the translation of an observed or imitated facial emotional expression into its internally felt emotional significance. Such results were interpreted by the authors as a mechanism that may mediate the understanding of the emotional state of others, thus contributing to empathy.

Overall, our results provide an encouraging exploratory paradigm to investigate the nature of emotional deficits in Schizophrenia that could be fruitfully coupled with neuroimaging studies aimed to investigate the neural substrate underpinning the deficits in rapid facial mimicry in patients suffering from schizophrenia.

### Conflict of interest statement

The authors declare that the research was conducted in the absence of any commercial or financial relationships that could be construed as a potential conflict of interest.
